# Endothelial-Ercc1 DNA repair deficiency provokes blood-brain barrier dysfunction

**DOI:** 10.1038/s41419-024-07306-0

**Published:** 2025-01-03

**Authors:** Cathrin E. Hansen, Davide Vacondio, Lennart van der Molen, Annika A. Jüttner, Wing Ka Fung, Manon Karsten, Bert van het Hof, Ruud D. Fontijn, Gijs Kooij, Maarten E. Witte, Anton J. M. Roks, Helga E. de Vries, Inge Mulder, Nienke M. de Wit

**Affiliations:** 1 Amsterdam UMC location Vrije Universiteit Amsterdam, Department of Molecular Cell Biology and Immunology, De Boelelaan 1117, 1081 HV Amsterdam, The Netherlands; 2 Amsterdam Neuroscience, Amsterdam UMC, Amsterdam, The Netherlands; 3 MS Center Amsterdam, Amsterdam UMC Location VU Medical Center, Amsterdam, The Netherlands; 4 Radboud University Medical Center, IQ Health science department, Nijmegen, The Netherlands; 5 Division of Vascular Medicine and Pharmacology, Department of Internal Medicine, Erasmus Medical Centre, Rotterdam, The Netherlands; 6 Amsterdam Institute for Infection and Immunity, Amsterdam UMC, Amsterdam, The Netherlands; 7 Amsterdam UMC location University of Amsterdam, Department of Biomedical Engineering and Physics, Meibergdreef 9, Amsterdam, The Netherlands; 8 Amsterdam Cardiovascular Sciences, Amsterdam UMC, Amsterdam, The Netherlands

**Keywords:** Experimental models of disease, Blood-brain barrier, Ageing, Senescence

## Abstract

Aging of the brain vasculature plays a key role in the development of neurovascular and neurodegenerative diseases, thereby contributing to cognitive impairment. Among other factors, DNA damage strongly promotes cellular aging, however, the role of genomic instability in brain endothelial cells (EC) and its potential effect on brain homeostasis is still largely unclear. We here investigated how endothelial aging impacts blood-brain barrier (BBB) function by using excision repair cross complementation group 1 (ERCC1)-deficient human brain ECs and an EC-specific *Ercc1* knock out (EC-KO) mouse model. In vitro, ERCC1-deficient brain ECs displayed increased senescence-associated secretory phenotype expression, reduced BBB integrity, and higher sprouting capacities due to an underlying dysregulation of the Dll4-Notch pathway. In line, EC-KO mice showed more P21^+^ cells, augmented expression of angiogenic markers, and a concomitant increase in the number of brain ECs and pericytes. Moreover, EC-KO mice displayed BBB leakage and enhanced cell adhesion molecule expression accompanied by peripheral immune cell infiltration into the brain. These findings were confined to the white matter, suggesting a regional susceptibility. Collectively, our results underline the role of endothelial aging as a driver of impaired BBB function, endothelial sprouting, and increased immune cell migration into the brain, thereby contributing to impaired brain homeostasis as observed during the aging process.

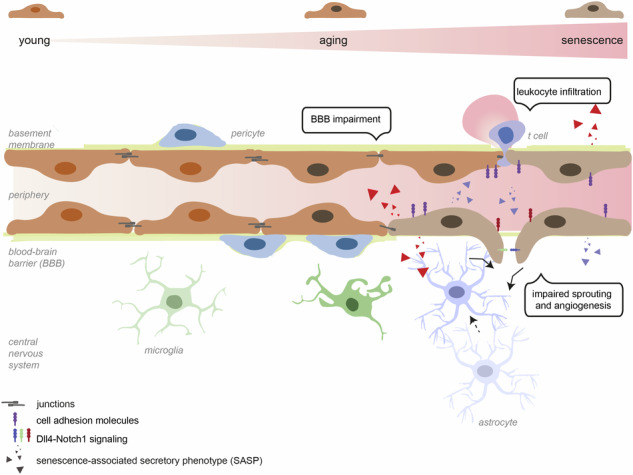

## Introduction

Brain endothelial cells (ECs) line the interior wall of cerebral microvasculature and establish the blood-brain barrier (BBB), which maintains the delicate homeostasis of the central nervous system (CNS). Brain endothelial tight junctions (i.e. Claudin-5) and adherens junction proteins (i.e. VE-cadherin) ensure the BBB-specific paracellular resistance, which prevents uncontrolled entry of blood components and infiltration of peripheral immune cells into the brain [1]. Additionally, BBB-specific transporters, such as the efflux transporter P-glycoprotein (P-gp) and major facilitator superfamily domain-containing protein 2a (Mfsd2a), regulate the metabolite exchange between CNS and periphery [2], ensuring optimal brain performance.

During aging, brain EC fitness and function are severely affected [3], leading to abnormal vascular responsiveness to cerebral blood flow and disruption of the BBB [4–7]. Aging-related alterations in BBB function include reduced integrity, altered transport mechanisms [8], and abnormal angiogenesis [9]. Physiological angiogenesis describes the multistep process of new vessel formation from the existing vasculature and is crucial to respond to the tissue’s oxygen needs [10]. In elderly, impaired angiogenesis and pathological vascular remodeling is suggested to contribute to microvascular rarefaction and potentially reduced tissue perfusion [9, 11–16]. Although dysfunction of brain ECs is recognized as a significant factor in the onset and progression of age-related neurodegenerative diseases such as stroke and different forms of dementia, including Alzheimer’s disease and vascular dementia, the underlying mechanisms remain elusive [17–21].

With age, cellular repair mechanisms are known to gradually deteriorate, leading to the accumulation of DNA damage and the advancement of cellular aging [22]. DNA damage response can induce aging via several mechanisms, including metabolic changes, transcriptional stress, and senescence [23, 24]. Cells can progress into senescence at the end-stage of their replicative capacity, marked by irreversible cell cycle arrest [25]. Senescent cells are metabolically active and acquire a cell-specific senescence-associated secretory phenotype (SASP) characterized by cytokine (i.e. Interleukins IL-6, IL-1β), chemokine (i.e. CXCL1, CXCL10), vasoactive mediator and growth factor (i.e. VEGF, TGF-β) production. Mouse models employing the deletion of the Excision repair cross complementation group 1 (*Ercc1*), a DNA repair endonuclease, have been successfully used to study human aging and senescence [23, 26]. *Ercc1* knock out mice (*Ercc1*^*Δ/-*^ KO) closely mimic human vascular aging by displaying increased vascular stiffness, extracellular matrix remodeling, and reduced vasodilator function [27, 28], thereby supporting the link between DNA damage and age-related vascular impairments. Nevertheless, it remains unclear how specifically endothelial aging affects BBB function.

In this study, we investigated the role of EC aging in BBB dysfunction and inflammation in vitro and in vivo. We report that ERCC1 deficiency in human brain ECs results in SASP expression, reduced BBB function, and enhanced endothelial sprouting via a dysregulation of the Dll4-Notch axis. In line, EC-KO mice demonstrate increased angiogenic marker expression as well as higher numbers of ECs and pericytes, specifically in the white matter (WM). EC-KO mice also display BBB leakage, glial reactivity at the vasculature and leukocyte infiltration in WM areas. Together, we suggest that senescent brain ECs accumulate during aging, thereby promoting BBB impairment and excessive sprouting, which in turn might contribute to the pathogenesis of neurodegenerative diseases.

## Materials and methods

### Animals

Endothelial-specific *Ercc1* KO animals were bred as described previously [27]. In brief, the Cre-loxP system was used to generate a conditional mouse model to knock out endonuclease *Ercc1* in ECs (B6.Cg-Tg(Tek-cre)12Flv/J, The Jackson Laboratory, Bar Harbor, USA). In the resulting litters, *Tie2*cre+/−:*Ercc1*fl/- mice have an *Ercc1* KO in ECs, where Cre-recombinase is active (from here on referred to as EC-KO mice). *Tie2*cre+/−:*Ercc1*fl/+ animals were used as wild type (WT) controls. Mice (male and female) were kept in individually ventilated cages, in a 12-h light/dark cycle with food and water *ad libitum*. Animals were euthanized around the age of 22 weeks by cardiac perfusion with ice-cold phosphate-buffered saline (PBS), after which brains were collected. Each brain was divided sagittally and one hemisphere snap-frozen, the other post-fixed in 1.6% paraformaldehyde (PFA) for 24 h followed by incubation in 30% sucrose for 24 hours. Sagittal brain slices (10 µm) were cut (CryoStar NX70, Thermo Fisher Scientific, Waltham, USA) and stored at −80 °C upon use. For details on the animals see Table 1.

**Table 1:** Details on WT and EC-KO mice.

Genotype	# mice	m/f
WT	14	6 / 8
EC-KO	11	8 / 3

*m* male, *f* female.

### Immunohistochemistry

Cryosections were defrosted, permeabilized, and blocked with 5% normal goat serum and 0.1% Triton-X100 in PBS (Sigma-Aldrich, Saint Louis, MO, USA), PBS only or Tris-buffered saline (TBS). Primary antibodies were incubated overnight at 4 °C. Tissue slides were incubated for 1 h at room temperature with secondary antibodies coupled to Alexa Fluor 488, 555 or 647 fluorophores (Molecular Probes, Eugene, OR, USA). The tissue slides were then counterstained with Hoechst (Molecular Probes, Eugene, OR, USA), embedded in Mowiol (in-house) mounting medium, and stored in the dark at 4°C until microscopic evaluation. For the 3,3′-diaminobenzidine (DAB) method, cryosections were fixed with 4% PFA (Sigma, Saint Louis, MI, USA) for 10 min and treated with 0.6% hydrogen peroxide for 10 min (Sigma, Saint Louis, MI, USA) to block endogenous peroxidase. The slides were then blocked with 5% human serum and 0.1% Triton X in PBS for 1 h. Next, the slides were incubated with primary antibody (IgG) overnight at 4 °C. The tissue was then treated with EnVision Dual Link System-HRP (Dako, Copenhagen, Denmark) solution for 30 min and DAB substrate solution (Dako, Copenhagen, Denmark) prepared according to the manufacturer’s instruction and applied for 5 min. Finally, the slides were stained with haematoxylin (made in-house) for 1 min. After rinsing in tap water for 5 min, the slides followed an alcohol/xylene series and were mounted in Entallan (Merck, Darmstadt, Germany). Antibody details are listed in Table 2.

**Table 2:** Primary antibody details.

Target	Host	Dilution	Ag retrival/fixation	Supplier	Cat.#	Use
α-SMA	Mouse	1:200	FF, none	Invitrogen	14-9760-82	IHC
CD8	Rabbit	1:200	Citrate	Abcam	ab183685	IHC
CD31	Mouse	1:100	4% PFA	Dako	M0823	ICC
CD45	Rat	1:200	Citrate	BD	553076	IHC
CLDN5	Rabbit	1:50	Methanol	Invitrogen	34-1600	IHC
CLDN5	Mouse	1:50	Methanol	Santa Cruz	sc-374221	ICC
COLLAGEN IV	Rabbit	1:200	FF, Acetone	Abcam	Ab5177	IHC
DLL4	Rat	1:100	4% PFA	R&D systems	207822	ICC
ERCC1	Rabbit	1:1000	none	Abcam	ab129267	WB
GAPDH	Mouse	1:1000	none	Proteintech	60004-1-lg	WB
GFAP	Rabbit	1:400	FF, none	DAKO	Z0334	IHC
yH2AX	Rabbit	1:500	4% PFA	Cell signaling	9718	ICC
IBA1	Goat	1:500	Citrate	Abcam	ab5076	IHC
ICAM1	Rat	1:50	FF, Methanol	Kind gift from Engelhardt Lab	In-house	IHC
IgG	Rabbit	1:200	4% PFA	Dako	P0260	IHC
LAMININ	Rabbit	1:500	FF, none	Novus	NB300-144	IHC
Lectin I-Rhodamine	-	1:250	None/citrate	Vector	RL-1102	IHC
MFSD2A	Rabbit	1:100	FF, none	Kind gift from Gu Lab	In-house	IHC
P21	Rat	1:100	FF, Acetone	abcam	ab107099	IHC
P2RY12	Rabbit	1:100	FF, none	Anaspec	55043 A	IHC
PDGFRβ	Rabbit	1:50	FF, none	Abcam	ab32570	IHC
MDR1A	Mouse	1:100	FF, none	Alexis	801-002-C100	IHC
SNAI2	Mouse	1:100	FF, acetone	Abcam	ab51772	IHC
VE-cad	Mouse	1:100	4% PFA	Santa Cruz	sc-9989	ICC
ZO-1	Rabbit	1:100	4% PFA	Zymed	61-7300	ICC

*FF* freshly frozen.

Immunocytochemistry (ICC) was performed similarly to IHC on human cerebral microvascular endothelial cells (hCMEC/D3) transduced with lentiviral constructs (see *Lentiviral short hairpin RNA knock down for ERCC1*). Shortly, cells were seeded in 8-well µ-slides (#80826, Ibidi, München, Germany) and fixed with 1.6% PFA, ice-cold methanol or acetone for 10 min at room temperature and permeabilized for 5 min using 0.05% Triton-X100 in PBS. Blocking, primary, and secondary antibody incubation occurred as described above. Nuclei were visualized using Hoechst and wells were filled with Mowiol before imaging. Antibody details are listed in Table 2.

### Microscopy and image acquisition

Images were acquired with wide-field imaging using the Olympus VS200 (Olympus, Tokyo, Japan) slide scanner, the Zeiss Axio Imager 2 (ZEISS, Jena, Germany), or confocal imaging using the Leica SP8 confocal microscope (Leica, Wetzlar, Germany) with a 60x or a 63x oil immersion objective, respectively. Regions of interest (ROI) were acquired as z-stacks of 4 or 8 µm and step sizes of 266 nm or 130 nm. For wide-field images, deconvolution was performed using Huygens Professional 21.10 software (Scientific Volume Imaging B.V., Hilversum, The Netherlands). NIS elements (version 5.30.03, Nikon Europe B.V., Amsterdam, The Netherlands), FIJI, and QuPath-0.2.3/-0.4.4 were used for automated and manual analysis. Three ROIs per brain region per animal were imaged in the white matter (WM; corpus callosum, dorsal fornix, and anterior commissure), the hippocampus (HC, supra and infra-pyramidal molecular layer), and the cortex (CRTX). For the vascular analysis, smooth muscle cells (SMCs) were identified by αSMA+ immunoreactivity (αSMA+, often PDGFRβ+ cells). Consequently, arterioles were defined as αSMA+ vessels and capillaries as αSMA-, PDGFRβ+ vessels with pericytes (PCs) (PDGFRβ+ cells) [29]. Capillary, arteriole ECs, PCs and SMCs are quantified as a % of total cells as well as their density as (PC/SMC+ vessel area / total vessel area) or coverage (PDGFRβ+/αSMA+ area/vessel area). Mean fluorescent intensity of PDGFRβ/ αSMA was measured within the vascular mask. Immune cell counts were performed blinded and manually by two researchers using QuPath-0.2.3/−0.4.4. Migrated immune cells were defined by their proximity to the vasculature as perivascular (still in contact with the abluminal side of the vessel marker) or parenchymal (minimal 10 µm distanced from the vessel). A fluorescent intensity threshold was applied on ICAM1 immunoreactivity to define positive and negative pixels. The percentage of ICAM1+ laminin+ objects over all laminin+ objects was quantified.

### Human brain endothelial cell culture

The immortalized hCMEC/D3 cell line was a kind gift provided by Prof. dr. IA Romero (Open University, Milton Keynes, UK) and Prof. dr. PO Coureaud (Université Paris Descartes, France) [30]. Cells were cultured from passages 29 to 39 in endothelial basal medium-2 (EBM-2) supplemented with 2.5% (v/v), heat-inactivated fetal bovine serum, growth supplement kit (#CC-3156, #CC-4147; Lonza, Basel, Switzerland), and 1% (v/v) penicillin-streptomycin (#15140-122; Gibco, Thermo Fisher Scientific, Waltham, USA). hCMEC/D3 cells were grown on bovine skin collagen I-coated culture flasks (#C5533; Sigma-Aldrich) until confluent unless stated otherwise. For culture, cells were maintained at 37°C and 5% CO_2_ and routinely screened for the presence of mycoplasma.

### Lentiviral short hairpin RNA knock down of *ERCC1*

Short hairpin RNAs were used to knock down *ERCC1* (shERCC1) expression in hCMEC/D3 as previously described [31–33]. Sub-confluent HEK 293T cells were co-transfected with the specific expression plasmids and packaging plasmids (pMDLg/pRRE, pRSV-Rev, and pMD2G) using calcium phosphate as transfection reagent. Infectious lentiviral particle-containing supernatant was collected after 48 h, concentrated using Amicon Ultra15 filters (UFC910024; Merck, Darmstadt, Germany) and stored at −80 °C upon further use. hCMEC/D3 cells were transduced at passage 30 by adding the concentrated supernatant 4–6 h after seeding and stable cell lines were selected 24 hours later using puromycin treatment (2 ng/ml, P7255; Sigma Aldrich). The knock down efficiency was assessed using quantitative real-time PCR (qRT-PCR) and Western blot. Constructs (TRCN0000049920) with 84% knock down efficiency were used for subsequent experiments. shERCC1 encodes for 5’- CAAGAGAAGATCTGGCCTTAT-3’. hCMEC/D3 cells transduced with lentivirus expressing non-targeting shRNA (NTC; SHC002, Sigma-Aldrich) were used as control cells. Transduced shERCC1 and NTC cells were used from passage (P) 1-7. The assessed gene expression in shERCC1 cells compared to NTC showed the same effect between P1 and P7, with varying effect sizes over the different passages.

### Induced pluripotent stem cell-derived brain pericytes

Human induced pluripotent stem cells (hiPSC) were differentiated into neural crest (NC)-derived brain pericytes (hiBPC) using previously published protocols [32, 34, 35]. Briefly, episomal hiPSC line (#A13700, Gibco, Thermo Fisher Scientific, Leusden, The Netherlands) was cultured in mTeSR Plus medium (STEMCELL Technologies, Vancouver, Canada) and grown on vitronectin-coated plates (Invitrogen, Thermo Fisher Scientific). HiPSCs were passaged as single cells, seeded onto Matrigel-coated plates (2 ×105 cells/cm^2^) and cultured for 5 days in NC induction medium, consisting of DMEM/F12 GlutaMAX™ (Gibco, Thermo Fisher Scientific), 1× B27 (Gibco, Thermo Fisher Scientific), 0.5% bovine serum albumin and 3 µM CHIR 99021 (Tocris, Bristol, United Kingdom). The resulting NC cells were seeded onto 0.1% gelatin-coated plates (2.5 × 104 cells/cm^2^) and cultured for an additional 5 days in pericyte medium (ScienCell, Carlsbad, CA, USA). iBPCs were characterized by immunocytochemistry (ICC) and RT-qPCR [32]. iBPCs were used between passages 2-4 in *spheroid-based sprouting experiments*.

### Electric cell-substrate impedance sensing (ECIS)

The transendothelial electrical resistance (TEER) of shERCC1 and NTC cells was assessed using the ECIS™ Model 1600R (Applied BioPhysics, Troy, NY) as previously reported [36, 37]. In short, cells were seeded at a density of 100.000 cells into 8W10 + ECIS arrays (#72040, Ibidi). Impedance was measured at multiple frequencies over a time course of 120 h. To quantify the maximum resistance [ohm], the data at 4000 Hz was normalized to the resistance at time before medium replacement.

### Scratch-wound and spheroid-based sprouting assay

For the scratch-wound assay, NTC and shERCC1 cells were grown to confluence and the scratch was induced diagonally with a plastic pipette tip. Cell migration was imaged for 22 h at ×10 magnification, bright field, at 37 °C with the Nikon Ti2 live cell imaging system (Nikon, Tokyo, Japan). Spheroid-based sprouting assays were performed as previously reported [32]. In brief, NTC and shERCC1 cells and iBPCs were re-suspended in a ratio of 20:1 in EGM-2 medium supplemented with 0.25% methylcellulose (4.000 cP, Sigma-Aldrich, Saint Louis, MO, USA). Cell suspension was seeded in a 24-well plate and flipped upside down. After 24 h, the spheroids were collected and re-suspended in 1.5 mg/ml collagen type-I rat tail mixture (Enzo science, Farmingdale, NY, USA) and re-plated in a 24-well plate upside down until complete polymerization. 30 min after polymerization, EGM-2 medium was administered and wells were incubated at 37 °C and 20% O_2_, 5% CO_2_ for 5 days. Images were taken using the Nikon LIPSI Ti2 confocal spinning disk imaging system (Nikon, Tokyo, Japan), ×10 objective, and adjusted for brightness/contrast in ImageJ. Sprouting number and length were analyzed using the ImageJ plugin NeuronJ [38].

### RNA isolation and real-time quantitative polymerase chain reaction (qRT-pcr)

Total RNA was extracted from mouse whole brain homogenates (WBH) using the RNeasy Lipid Tissue Mini Kit (#174804, Qiagen) and from hCMEC/D3 using TRIzol (#15596-018, Thermo Fisher Scientific). RNA quantity was assessed by nanophotometer (Implen, Westlake Village, USA). The High-Capacity cDNA Reverse Transcription Kit (#4368813, Thermo Fisher Scientific) was used to synthesize cDNA and transcripts of interest were detected with SYBR Green (#4309155, Thermo Fisher Scientific) using the QuantStudio™ 3 Real-Time PCR System (#A28567, Thermo Fisher Scientific). Expression was normalized to housekeeping genes *β-actin* (WBH) and glyceraldehyde 3-phosphate dehydrogenase (*GAPDH*; hCMEC/D3) using the 2−^ΔΔ^CT relative quantification method. Primer sequences are summarized in Supplementary Table 1.

### Nuclear fractionation and western blot

hCMEC/D3 cells were washed with cold PBS and lysed on ice with cell lysis buffer (Cell Signaling Technology, Boston, MA, USA) containing protease and phosphatase inhibitors (Roche, Almere, The Netherlands, and Cell Signaling Technology, Boston, MA, USA, respectively). Nuclear fractions were isolated using the NE-PER extraction kit (Thermo Fisher Scientific), following the manufacturer’s instructions. All samples were diluted in Laemmli buffer (2x) (BioRad Hercules, CA, USA) (65.8 mM Tris-HCl, pH 6.8, 2.1% SDS, 26.3% (w/v) glycerol, 0.01% bromophenol blue) and heated to 95 °C for 3–5 min. Lysates were separated on SDS-PAGE followed by transfer to nitrocellulose for immune blot analysis. Blots were blocked with blocking buffer (Licor, Lincoln, USA) for 1 h at room temperature. Subsequently, membranes were incubated in blocking buffer containing 0.1% Tween-20 with primary antibodies (Table 2) overnight at 4°C and detected and quantified by incubation with IRDye secondary antibodies (1 h, room temperature) (LI-COR) and imaged by Azure Sapphire Biomolecular Imager (Azure Biosystems, Inc, Sierra CT, Dublin, CA, USA). Original Western blots are depicted in Supplementary Fig. 1b.

### Statistical analysis

All analyses were performed blinded and data are plotted as box plots with median ± quartiles and whiskers extending to minimum and maximum values. Statistical tests were performed using GraphPad Prism v9 (GraphPad Software, La Jolla, USA). We used the Shapiro–Wilk test for data normality. For comparing two experimental groups, two-tailed Student’s *t* test was used and non-parametric data was analyzed by the Mann–Whitney test. Statistical significance was set at *p* < 0.05 and nominal p-values are reported throughout the manuscript. For multiple Student’s *t* test Benjamini–Hochberg correction was performed (*q* = 10%) and the *q*-values can be found in Supplementary Table 2 and 3. Of note, all significantly different genes survived FDR correction. Test details are indicated in the corresponding figure legend. For the creation of the gene expression heat map, we used the web-based tool MetaboAnalyst (http://www.metaboanalyst.ca, accessed: 10/07/2023).

### Ethics approval and consent to participate

All animal procedures were performed at the Erasmus Laboratory Animal Science Center following the guidelines from Directive 2010/63/EU of the European Parliament on the protection of animals used for scientific purposes and approved by the National Animal Care Committee and the administration within Erasmus University Medical Center Rotterdam (protocol number 118-13-03). All methods were performed in accordance with the relevant guidelines and regulations.

## Results

### ERCC1 deficiency induces BBB impairment in brain ECs

**Fig. 1:**
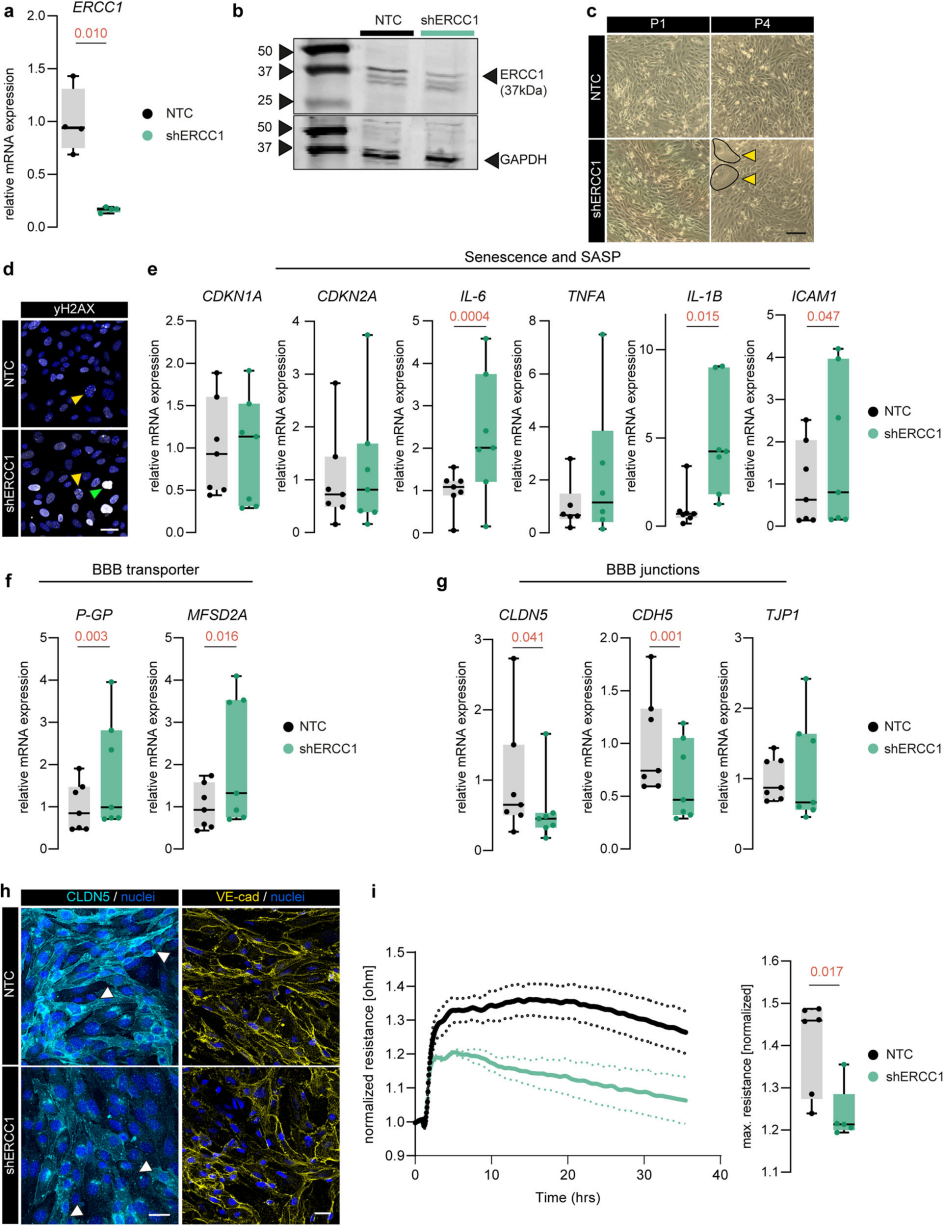
ERCC1 deficiency induces cellular aging phenotype and impairs BBB function in brain ECs. **a** mRNA levels of *ERCC1* in non-targeting control (NTC) and ERCC1 knock down (shERCC1) human brain ECs (hCMEC/D3), *n* = 4. **b** Nuclear fraction of ERCC1 in NTC and shERCC1 hCMEC/D3 evaluated by Western blot and GAPDH used as a reference protein. **c** Representative image of shERCC1 and NTC cells at passage one (P1) and four (P4) after virus transduction. Enlarged cells are encircled in black; scale bar: 200 µm. **d** Representative images of phosphorylated yH2AX in shERCC1 and NTC cells (yH2AX, white); scale bar: 25 µm. Yellow arrowheads indicate yH2AX foci and green arrowhead yH2AX pan-expression. **e** mRNA expression of senescent and SASP targets (*P21, P16, IL-6, TNFA, IL-1B, ICAM1*) in NTC and shERCC1 cells, *n* = 4. **f** mRNA expression of BBB transporters *P-GP* and *MFSD2A* in shERCC1 and NTC cells (*n* = 7). **g** mRNA expression of BBB junction marker *CLND5*, *CDH5* and *ZO-1* in shERCC1 and NTC cells (*n* = 7). Data have been normalized to *GAPDH* and presented as fold change to NTC values. Each data point represents the mean of a single experiment performed in triplicates. **h** Representative images of CLDN5 (white arrowheads indicate junctional immunoreactivity) and VE-cad in shERCC1 and NTC cells (scale bar: 25 µm). **i** Transendothelial electrical resistance shown over time and quantification of maximal resistance (box plot) in shERCC1 and NTC cells (*n* = 5,6). Data is shown as box plots with median ± quartiles; whiskers extend to minimum and maximum. Statistical comparison of two groups was performed using (paired) two-tailed Student’s t-test for normally distributed data, or the Wilcoxon-test/ Mann-Whitney test for non-normally distributed data. Exact *p*-values are reported and statistical significance is set at *p* < 0.05.

To investigate the effect of aging on brain EC function in vitro, we generated an accelerated aging model by reducing the expression of Ercc1 (shERCC1) in a human brain EC cell line (hCMEC/D3). shERCC1 cells expressed less *ERCC1* mRNA (83%, *p* = 0.010, Fig. 1a) and showed less ERCC1 protein (Fig. 1b, Supplementary Fig. 1b) compared to the non-targeting control cells (NTC). With increasing passage (P) number, shERCC1 cells adopted an enlarged cell size (encircled, Fig. 1c), which is characteristic of senescent cells [39]. As a measure of DNA damage, we evaluated the phosphorylation of histone H2AX (yH2AX) [40]. shERCC1 cells showed yH2AX foci (yellow arrowhead) and some pan-nuclei H2AX phosphorylation (green arrowhead) (Fig. 1d). Among the tested senescence and SASP markers, we found a significant increase in *IL-6* (*p* = 0.004), *IL-1B* (*p* = 0.016) and intercellular adhesion molecule 1 (*ICAM1*) (*p* = 0.047) mRNA expression in shERCC1 cells compared to NTC, while no significant differences were observed in *TNFA*, CDKN1A and CDKN2A expression (Fig. 1e).

Next, we investigated the expression of BBB transporters and junction components upon silencing Ercc1. shERCC1 cells showed an increased expression of the transporters *PGP* (*p* = 0.003) and *MFSD2A* (*p* = 0.016) compared to NTC cells (Fig. 1f). Junctional markers like claudin5 (Clnd5) and VE-cadherin (VE-Cad, *CDH5*) were decreased in shERCC1 cells, both in their RNA expression (*CLDN5;* *p* = 0.0156, *CDH5;* *p* = 0.0010) and protein level (Fig. 1g, h). Zona occludens-1 (ZO-1, *TJP1*) did not differ between conditions (Fig. 1g, Supplementary Fig. 1a). In line with decreased CLDN5 and VE-Cad levels, shERCC1 cells displayed a significantly reduced barrier resistance compared to NTC cells (*p* = 0.017) (Fig. 1i). Together, these results indicate that ERCC1 knock down induces DNA damage accumulation and BBB dysfunction in brain ECs.

### ERCC1 deficiency enhances migration and sprouting of brain ECs in vitro

**Fig. 2:**
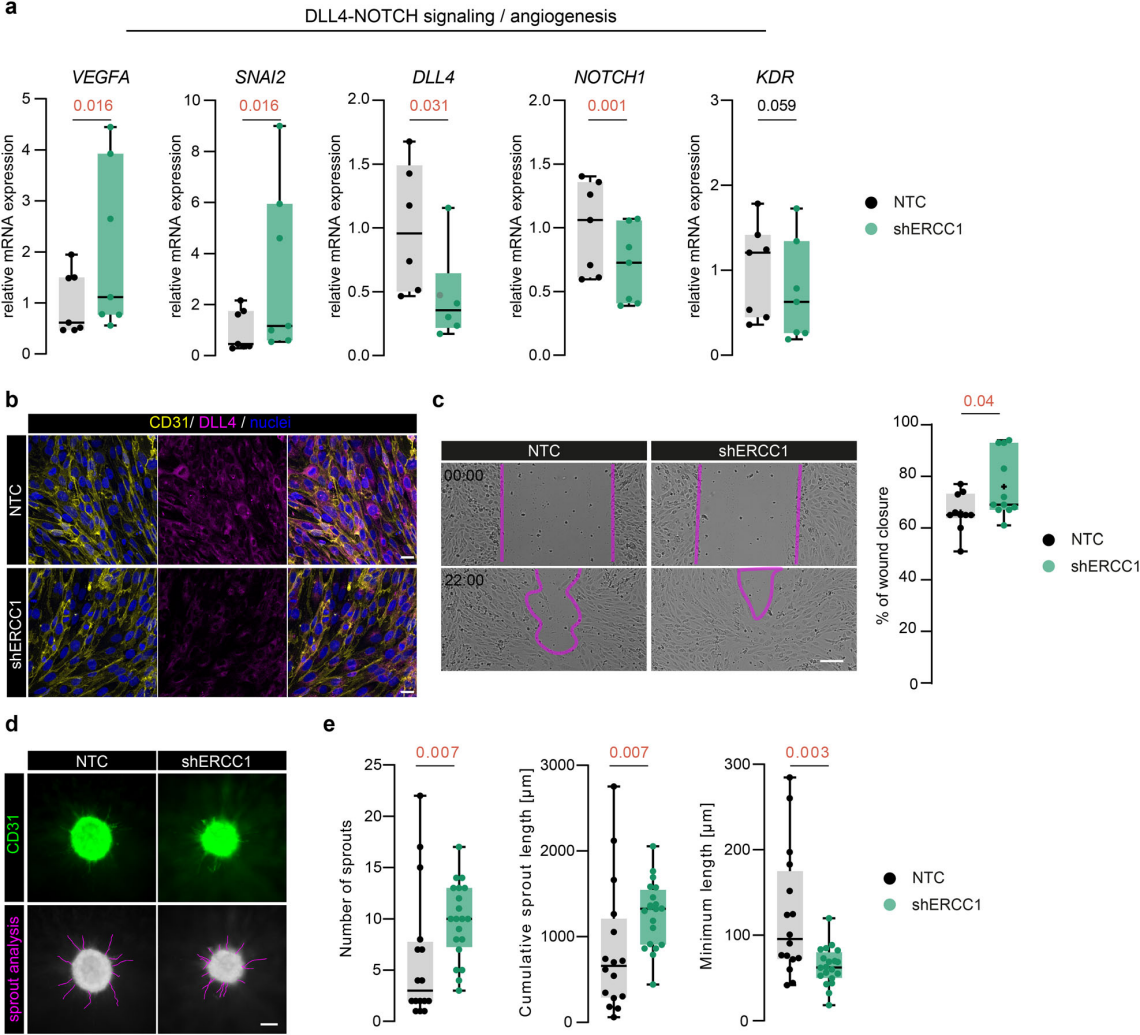
shERCC1 cells show enhanced endothelial migration and sprouting. **a** mRNA expression of angiogenic markers *VEGFA*, *SNAI2*, *DLL4*, *NOTCH1* and *KDR* in shERCC1 and NTC cells (*n* = 4). **b** Representative images of CD31 and DLL4 expression in shERCC1 and NTC cells. **c** Representative images of scratch-wound assay at *t* = 0 and *t* = 22 h in shERCC1 and NTC cells. The pink outline indicates the scratch borders. **d** Representative images of CD31 in sprouting shERCC1 and NTC cells with the manual analysis of sprouts marked in pink (scale bar: 150 µm). **e** Quantification of total number of sprouts, cumulative sprout length, and minimum sprout length per cell type (*n* = 16–20). Each dot represents a biological replicate and for the qPCR an average of technical triplicates, presented as box plots with median ± quartiles; whiskers extend to minimum and maximum. Statistical comparison of two groups was performed using (paired) two-tailed Student’s *t* test for normally distributed data, or the Mann–Whitney test for non-normally distributed data. Exact *p*-values are reported and statistical significance is set at *p* < 0.05.

The reduction in BBB markers such as Cldn5 can underlie a (transient) loss of EC identity, which has been associated, among others, with angiogenesis and vascular remodeling [41]. Thus, we evaluated the delta like canonical notch ligand 4 (Dll4)-Notch1 axis, which is fundamental in the regulation of EC sprouting angiogenesis [42]. *NOTCH1* (*p* = 0.0009) and *DLL4* (*p* = 0.0313) mRNA expression were decreased in shERCC1 cells compared to NTCs (Fig. 2a), and DLL4 density was reduced in shERCC1 cells (Fig. 2b). Next, we assessed the mRNA expression of *VEGFA* and kinase insert domain receptor (*KDR*, gene encoding vascular endothelial growth factor receptor 2), which are pivotal in the regulation of the Dll4-Notch1 pathway. We observed a significant increase of *VEGFA* (*p* = 0.016) and a decreasing trend for *KDR* (*p* = 0.059) in shERCC1 cells compared to NTCs (Fig. 2a). Lastly, we assessed the mRNA expression of *SNAI2*, a transcription factor which has been shown to directly regulate DLL4 expression in ECs [43]. We report a significant increase in *SNAI2* mRNA expression (*p* = 0.016) in shERCC1 cells compared to NTC cells (Fig. 2a).

To functionally assess the dysregulated Notch pathway in shERCC1 cells, we performed a scratch-wound assay and a sprouting assay. shERCC1 cells closed the scratch significantly faster than the NTC cells (Fig. 2c). Further, shERCC1 cells showed a significant increase in the number of sprouts (*p* = 0.007) and cumulative sprout length (p = 0.007) compared to NTC cells (Fig. 2d, e). The minimum length of the sprouts was significantly decreased in shERCC1 cells compared to NTC cells (*p* = 0.003) (Fig. 2e). No change was observed in the mean or maximum sprout length (Supplementary Fig. 2a). Together these data indicate a dysregulated Dll4-Notch1 axis in shERCC1 cells, which may explain their impaired angiogenic capacity.

### EC-specific *Ercc1* ko mice show senescence and increased BBB transporters in white matter tissue

**Fig. 3:**
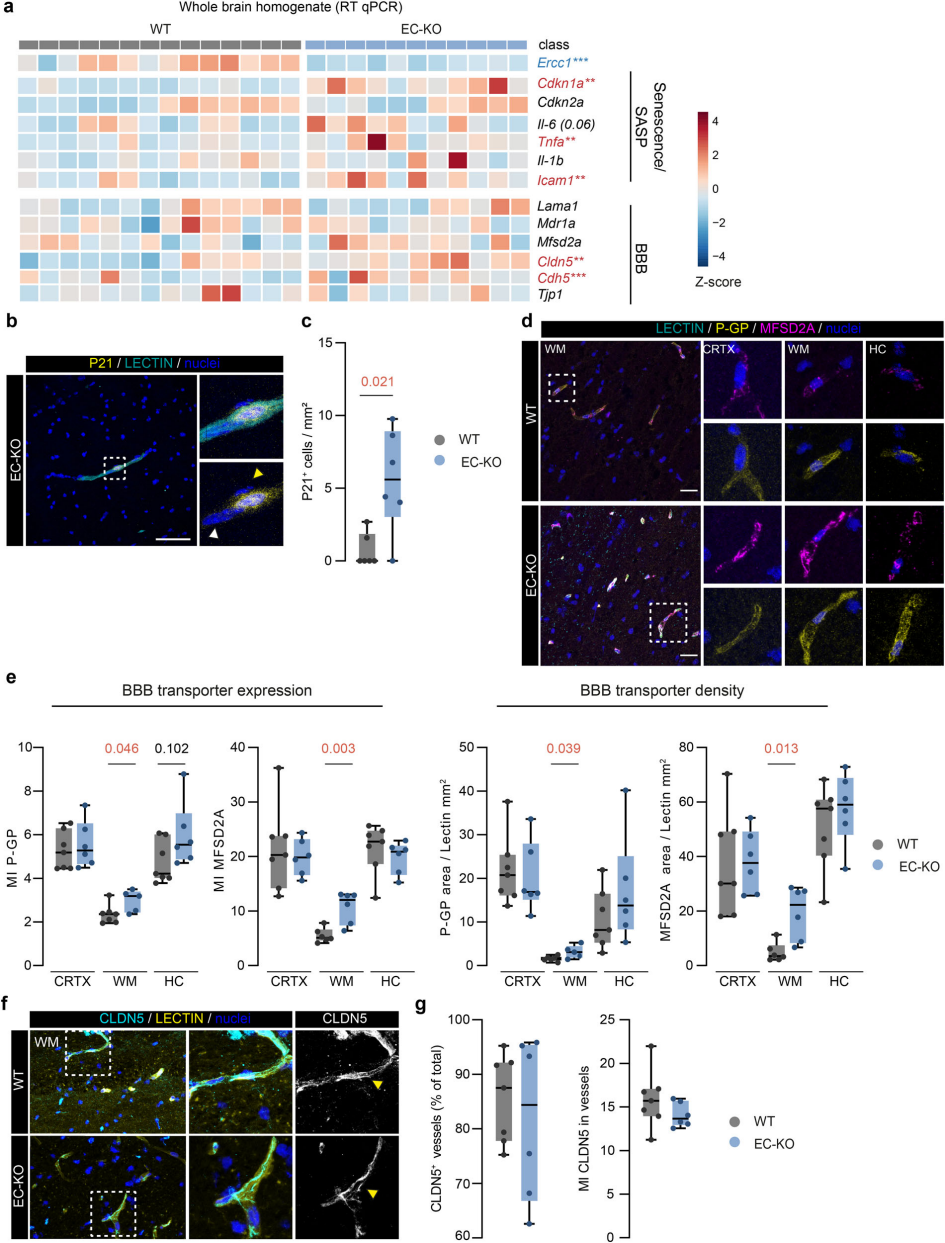
EC-KO mice display increased number of P21^*+*^ cells and BBB transporters specifically in the white matter. **a** Heatmap visualizes gene expression profile of WBH comparing EC-KO mice with WT mice. Target categories comprise senescence and BBB markers (*n* = 11–14). **b** Representative image of P21 (senescent cell identifier) and Lectin immunoreactivity in EC-KO mice brain tissue; yellow arrowhead indicates P21^*+*^ nucleus; white arrowhead indicates P21^-^ nucleus (scale bar: 50 µm). **c** Quantification of total vascular P21^*+*^ cells per mm^2^ in WT and EC-KO (*N* = 6). **d** Representative images of MDR1A and MFSD2A reactivity in cortex (CRTX), white matter (WM), and hippocampus (HC) in WT and EC-KO brains (scale bar: 50 µm). **e** Quantification of mean fluorescent intensity (MI) of MDR1A and MFSD2A in Lectin^+^ area (transporter expression) and transporter area normalized to Lectin area (transporter density) (*n* = 6). **f** Representative images of CLDN5 reactivity in WM of WT and EC-KO mice (scale bar: 25 µm). **g** Quantification of CLDN5^+^ vessels (CLDN5^+^, Lectin^+^ objects) and MI of CLDN5 in Lectin^+^ area (*n* = 6–7). Data is shown as box plots with median ± quartiles; whiskers extend to minimum and maximum. All data have been statistically tested by unpaired student-t test with Welch’s correction when the variance of the groups was significantly different or Mann–Whitney test for non-parametric datasets. Exact p-values are reported and statistical significance is set at *p* < 0.05 (red).

To study the impact of EC-specific accelerated cellular aging on brain homeostasis in vivo, we utilized EC*-*KO mice [27]. First, we examined the senescence profile of EC-KO mice compared to WT mice by using multiplex qPCR on whole brain homogenates (WBH) (Fig. 3a). *Ercc1* mRNA expression was reduced in EC-KO compared to WT brains (*p* = 0.0001). The mRNA expression of the senescence markers *Cdkn1a* (encoding P21) (*p* = 0.002), *Tnfa* (*p* = 0.002), and *Icam1* (*p* = 0.004) was increased in EC-KO brains, and *Il-6* (*p* = 0.06) showed a similar trend, while C*dkn2a* (encoding P16) and *Il-1b* did not differ between genotypes (Fig. 3a). Immunohistochemical analysis also showed enhanced levels of P21 in EC-KO mice (*p* = 0.021) compared to WT mice which co-localized with Lectin, an endothelial cell marker (*p* = 0.021; Fig. 3b, c).

To evaluate the properties of the BBB in the EC-KO mice, we next investigated the mRNA expression of BBB-associated markers in WBH. We found a significant increase in *Cldn5* and *Cdh5* mRNA expression in EC-KO mice compared to WT (*p* = 0.009 and *p* = 0.0009, respectively), while *Lama1, Tjp1, Mdr1a* (encoding P-GP), and *Mfsd2a* were unchanged (Fig. 3a). We then examined P-GP and MFSD2A levels in the different brain regions of EC-KO and WT using immunohistochemistry (Fig. 3d). We found an increase of P-GP (*p* = 0.046) and MFSD2A (*p* = 0.003) immunoreactivity (fluorescent mean intensity (MI)) in the white matter (WM) and a trend towards increased P-GP expression in the hippocampus (HC) (*p* = 0.102) of EC-KO mice compared to WT (Fig. 3e). Concomitantly, we found higher transporter coverage (reactivity area/Lectin area) of the vasculature (P-GP (*p* = 0.039); MFSD2A (*p* = 0.013)) in the WM of EC-KO mice. No differences were found in the cortex (CRTX) or HC. Focusing from now on the WM, we analyzed CLDN5 levels via immunohistochemistry. No differences were observed when comparing the number of CLDN5^+^ vessels and the MI of CLDN5 within the vasculature between EC-KO and WT (Fig. 3f, g). In summary, endothelial specific *Ercc1*-mediated aging increases P21^+^ cells and BBB transporter levels specifically in the WM.

### EC-KO mice increase angiogenic marker expression and show BBB leakage

**Fig. 4:**
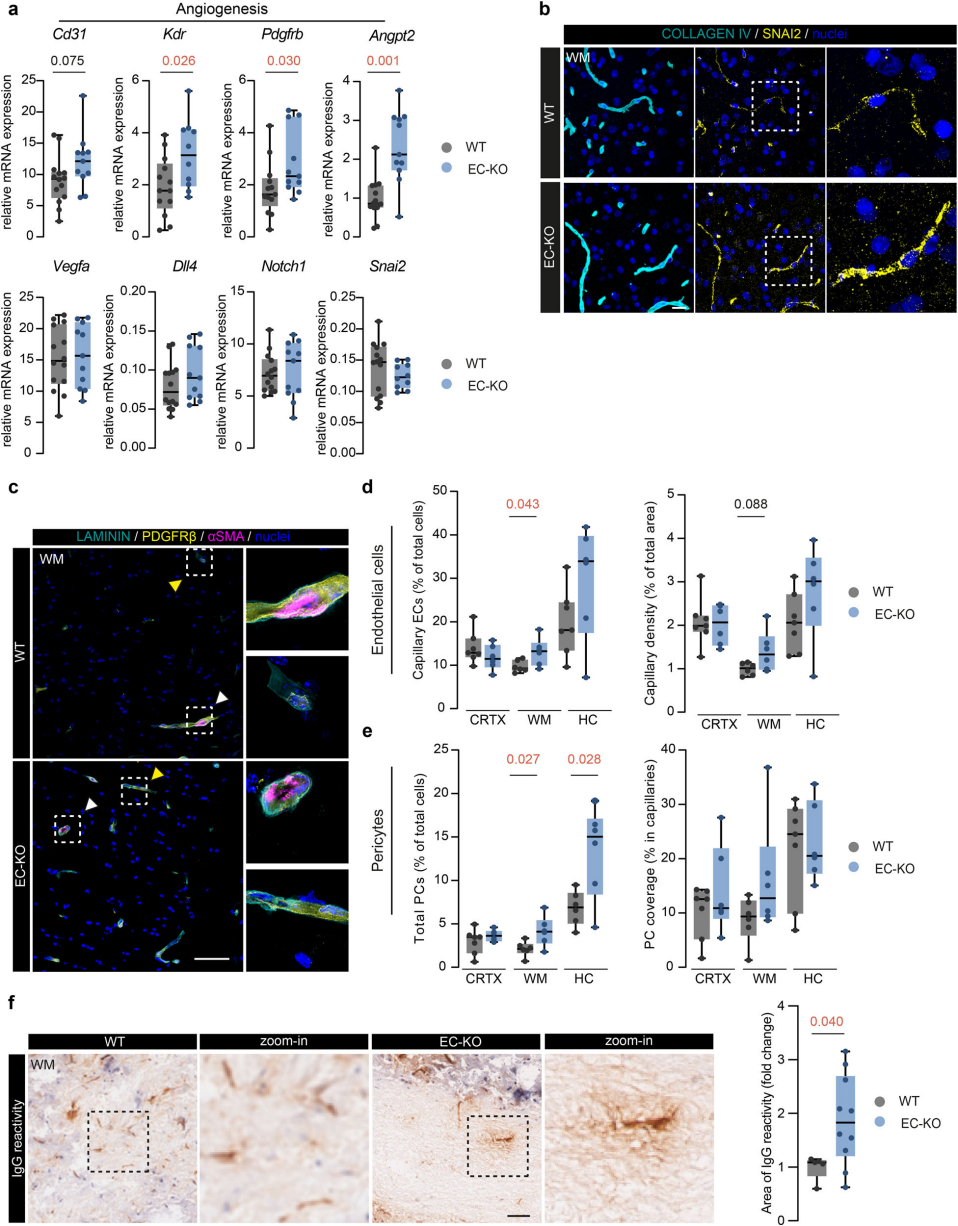
EC-specific Ercc1 deficiency induces angiogenic markers and BBB leakage in vivo. **a** mRNA expression of *Cd31, Vegfa, Dll4, Notch1, Kdr, Snai2, Pdgfrb, and Angpt2* in WBH are plotted as box plots to visualize effect size in EC-KO and WT mice (*n* = 11–14). **b** Representative image of COLLAGEN IV and SNAI2 immunoreactivity in the WM of EC-KO and WT mice. **c** Representative images of LAMININ, PDGFRβ and αSMA immunoreactivity of WM brain tissue in WT and EC-KO mice; white arrowheads indicate αSMA^*+*^ vessels (arteriole, upper panel), yellow arrowheads indicate PDGFRβ^+^, αSMA^*-*^ vessels (capillary, lower panel) (scale bar: 50 µm). **d**, **e** Quantification of capillary ECs and capillary density as well as pericyte number and coverage in EC-KO and WT mice. **f** Representative images of IgG immunoreactivity in EC-KO and WT mice and semi-quantification of IgG^+^ area (*n* = 5–10; scale bar: 20 µm). Data is shown as box plots with median ± quartiles; whiskers extend to minimum and maximum. All data have been statistically tested by unpaired Student’s *t* test with Welch’s correction when the variance of the groups was significantly different or Mann–Whitney test for non-parametric datasets. Exact p-values are reported and statistical significance is set at *p* < 0.05.

Following our findings in shERCC1 cells (Fig. 2), we studied angiogenesis-related markers in the WBH of EC-KO and WT mice. We observed an increase of *Kdr* (*p* = 0.026), platelet-derived growth factor receptor beta (*Pdgfrb)* (*p* = 0.030), angiopoietin2 (*Angpt2*) (*p* = 0.001) and a positive trend for *Cd31* (*p* = 0.075) in EC-KO mice compared to WT (Fig. 4a)*. Vegfa, Dll4, Notch1* and *Snai2* mRNA did not change between the experimental groups, but we observed an increase in SNAI2 on protein level in the WM vasculature of EC-KO mice compared to WT (Fig. 4b).

Since we found an increase in angiogenic markers in EC-KO mice, we next investigated potential changes in the vascular architecture in EC-KO and WT mouse brains. We used a triple immunostaining with PDGFRβ, alpha-smooth muscle actin (αSMA), and LAMININ, to discriminate capillaries from arterioles (Fig. 4c, Supplementary Fig. 3a) [44]. Capillary ECs (% of total cells) were increased in the WM of EC-KO compared to WT mice (*p* = 0.043), but not in CRTX or HC (Fig. 4d). In contrast, arterial ECs (% of total cells) did not differ between genotypes in all regions (Fig. 4d, Supplementary Fig. 3b). The capillary density (area of αSMA-, PDGFRβ+ vessels/total area) in the WM of EC-KO mice showed an increasing trend (*p* = 0.088), while overall vascular density and arterial density (area of αSMA+, PDGFRβ+ vessels/total area) did not change between EC-KO in WT in all three brain regions (Fig. 4d, Supplementary Fig. 3c). Finally, PCs (% of total cells) were significantly higher in the WM (*p* = 0.027) of EC-KO mice as well as in the HC (p = 0.028). The PC coverage (PDGFRβ+ area/laminin area) of the endothelium was unaffected (Fig. 4e). No differences were detected in smooth muscle cells (SMCs) and SMC vessel coverage, nor in the mean expression levels (MI) of PDGFRβ and αSMA between EC-KO and WT (Supplementary Fig. 3d, e). Lastly, we assessed if the vascular changes resulted in BBB leakage. We found an increased IgG reactivity in the WM of EC-KO animals compared to WT (*p* = 0.040) (Fig. 4f), while IgG reactivity did not differ between genotypes in the gray matter (average CRTX and HC) (Supplementary Fig. 3f). Taken together, these data indicate that EC-KO mice display microvascular changes in the WM which results in local BBB leakage.

### EC-KO mice display an inflamed BBB and immune cell infiltration in the white matter

**Fig. 5:**
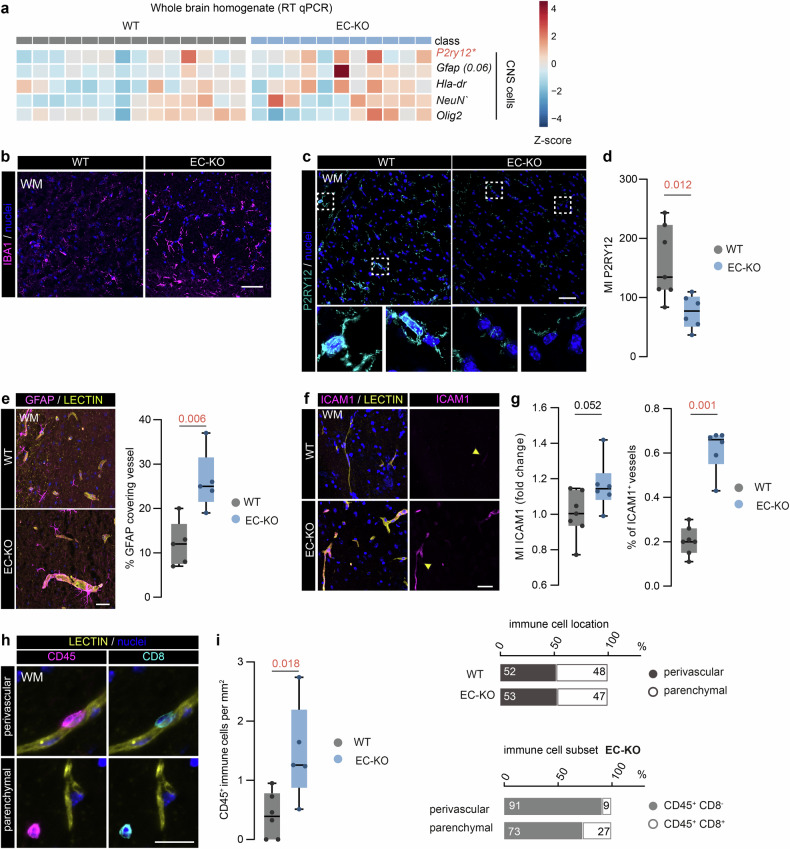
Enhanced vascular ICAM1 expression and immune cell infiltration in the white matter of EC-KO mice. **a** Multiplex qPCR mRNA on WBH comparing EC-KO and WT mice (*n* = 11–14). **b** Representative images of IBA1 immunoreactivity in WM of EC-KO and WT mice (scale bar: 50 µm). **c** Representative images of P2RY12 immunoreactivity in WM of EC-KO and WT mice (scale bar: 50 µm). **d** Quantification of P2RY12 MI in EC-KO and WT mice (*n* = 6–7). **e** Representative images and quantification of GFAP^+^ area covering the vessel (% GFAP in Lectin^+^ area) in WM of EC-KO and WT mice (scale bar: 25 µm). **f** Representative images of ICAM1^*+*^vessels in EC-KO and WT mice (scale bar: 30 µm). **g** Quantification of ICAM1^*+*^ vessels and vascular ICAM1 expression in both WT and EC-KO WM brain tissue (*n* = 5–7). **h** Representative images of CD45, CD8, and Lectin immunoreactivity presenting both perivascular (top panel) and parenchymal (lower panel) location of peripheral immune cells (scale bar: 20 µm). **i** Quantification of CD45^+^ immune cells per mm^2^ in EC-KO and WT mice. Data is shown as box plots with median ± quartiles; whiskers extend to minimum and maximum. All data have been statistically tested by unpaired Student’s *t* test with Welch’s correction when the variance of the groups was significantly different or Mann–Whitney test for non-parametric datasets. Exact *p*-values are reported and statistical significance is set at *p* < 0.05 (red).

Based on the observed IgG leakage, specifically in the WM of EC-KO mice, we next focused on the possible presence of local inflammation. In the whole brain lysates, we found a significant increase of *P2ry12* mRNA (*p* = 0.027), a homeostatic marker for microglia, and a similar trend for *Gfap* (*p* = 0.06), a marker for reactive astrocytes (Fig. 5a). In WM tissue, we observed more IBA1^*+*^ cells in EC-KO mice compared to WT (Fig. 5b). Furthermore, we found a decreased P2RY12 levels in the WM of EC-KO mice (*p* = 0.012) compared to WT (Fig. 5c, d), which may indicate microglia activation. Of note, the GFAP-vessel co-localization was higher in the WM of EC-KO (P = 0.006) compared to WT. (Fig. 5e, Supplementary Fig. 4a). Next, we analyzed BBB inflammation by the vascular expression of ICAM1. EC-KO mice displayed more ICAM1^+^ vessels (*p* = 0.001) and a trend towards higher vascular ICAM1 levels (*p* = 0.052) in the WM compared to WT (Fig. 5f, g). Using CD45 to identify leukocytes and CD8 for cytotoxic CD8^+^ T cells specifically (Fig. 5h), we found an increased density of immune cells in the WM of EC-KO mice (*p* = 0.018) compared to WT. Almost half of the cells were in the parenchymal tissue similar in WT and EC-KO brains (Fig. 5i). 27% of parenchymal cells were CD8^+^ T cells in the EC-KO mice compared to 9% of the perivascular cells (Fig. 5i). In the WT brains, all parenchymal Cd45^+^ cells were positive for Cd8 (total count: 2 cells), while all perivascular cells were Cd8^-^ (Supplementary Fig. 4b). In summary, our findings show that endothelial aging coincides with BBB inflammation and increased peripheral immune cells migration into the brain, highlighting a key role of endothelial cells in CNS aging and subsequent inflammation, specifically in the WM.

## Discussion

Preclinical and clinical studies indicate that aging is a critical factor inducing endothelial dysfunction [3, 45, 46]. In line, brain endothelial dysfunction is frequently found during healthy brain aging as well as in neurological disorders such as stroke and Alzheimer’s disease [47–50]. However, the role of brain endothelial aging and senescence in BBB impairment remains largely unknown. In this study, we evaluated the consequences of ERCC1 deficiency, a model for accelerated aging, in brain ECs in vitro and in vivo. We show that ERCC1-deficient brain ECs display reduced BBB integrity, increased transporter expression, and more endothelial sprouting. We validated our in vitro findings in EC-KO mice, which display a higher expression of angiogenic genes and more capillary ECs and pericytes, specifically in the WM. Furthermore, the WM of the EC-KO animals demonstrates IgG leakage and increased glial cell reactivity near the vasculature, which coincided with immune cell infiltration in the brain parenchyma. Together, our work highlights the effect of EC aging on BBB dysfunction, angiogenesis and local inflammation.

In this study, shERCC1 display classical hallmarks of DNA damage and cellular aging including γH2AX phosphorylation and increased SASP component expression (i.e. *IL-1B*, *IL-6*, *VEGFA*). Conversely, the expression of *CDKN1A* (P21) and *CDKN2A* (P16), known senescence markers [51], was unaffected in shERCC1 cells. SASP and P21/P16 elevation are not always concomitant [52, 53], and the presence of the latter is not a pre-requisite for cell aging as exemplified by studies in post-mitotic cardiomyocytes [54, 55]. Furthermore, in our set-up, the high proliferative capacity of hCMEC/D3 cells combined with ERCC1 deficiency, may highlight cellular aging features directly associated with DNA damage-related cell stress, while overshadowing the P21/P16 expression present in the few senescent cells with halted cell cycle. Lastly, VEGF, highly expressed in the shERCC1 cells, is known to negatively regulate P21/P16 expression [56], which may explain the similarity with NTC cells. The cellular stress of shERCC1 cells is also accompanied by a reduced expression of Cldn5 and VE-cad, resulting in impaired barrier integrity, as seen during aging in vivo and in vitro [57, 58]. Together, these results substantiate the role of DNA damage in inducing cell aging, and highlight the effect of brain EC aging on BBB function.

In vivo, EC-KO mice displayed increased vascular P21 expression compared to WT animals. However, not all brain ECs were P21^+^, which aligns with previous reports on this model suggesting partial efficiency of the Cre-lox system in deleting *Ercc1* [27, 59, 60]. P21 expression is increased in the brain endothelium of elderly compared to young individuals [59, 61] and MRI studies positively associate age and enhanced BBB permeability in healthy elderly suggesting the possible effect of aging on BBB dysfunction [62–64]. Of note, in EC-KO mice both peripheral and brain ECs are affected by Ercc1 deletion. We cannot exclude partial effects on the BBB arising from the dysfunction of peripheral vasculature associated with aging. In our study, EC-KO mice displayed increased IgG leakage, possibly underlying reduced BBB resistance resulting from EC aging, which is in line with our in vitro findings on BBB dysfunction. Furthermore, we observed an increase in *Cldn5* and *Cdh5* expression in the whole brain homogenate of EC-KO animals compared to WT, but no changes in Cldn5 expression in the WM vessels. Interestingly, we show an increased number of brain EC in WM capillaries, which could partially explain the increase in junction mRNA expression. It is important to clarify that we used brain lysates to identify potential targets for initial investigation, which were validated with region- and cell-specific techniques, such as immunohistochemistry. In sum, our findings present an in vivo model for accelerated vascular aging, which recapitulates some of the features, including reduced BBB integrity, observed during healthy cerebrovascular aging in humans.

In EC-KO mice, the increased number of ECs in WM brain capillaries was concomitant with an enhanced expression of angiogenic markers (*Angpt2*, *Kdr*), suggesting increased EC sprouting. Furthermore, we observed a general increase in vascular SNAI2 expression in the WM on EC-KO animals. High Snai2 expression has been previously shown to directly impair the Dll4-Notch1-axis, resulting in angiogenesis characterized by dysfunctional vessels [43]. Similarly, shERCC1 cells displayed increased *SNAI2* and reduced *DLL4* and *NOTCH1* expression together with increased sprout number. These findings may indicate a reactivation of the angiogenic program in the WM of EC-KO mice sustained by Snai2. In line with our findings, previous studies found increased angiogenic markers (i.e. Angpt2) in brain ECs isolated from the corpus callosum of aged mice compared to younger animals [65]. Interestingly, in our study we found the major changes in the WM of EC-KO mice, while the CRTX and HC seemed to be less affected, suggesting a regional susceptibility to EC aging. In humans, WM has been previously shown to be more susceptible to age-related pathologies including vascular dementia [66, 67]. A possible explanation may lie in the inherent lower capillary density of the WM, which makes this area more sensitive to hypoxic insults, a known trigger for angiogenesis via different pathways including Snai2 upregulation [50, 65, 66, 68–71]. Eventually, studies in aged mice and elderly show vessel rarefaction and reduced vessel length, which may be the result of dysfunctional angiogenesis [11, 72–76]. Together our data suggest that DNA damage in brain ECs may sustain dysfunctional angiogenesis via dysregulated Dll4-Notch1 signaling, and that the WM is more susceptible to this process. However, more research is warranted to fully comprehend the mechanisms underlying brain vasculature maintenance and remodeling during aging.

With age, a decrease in BBB transporter expression is observed [8, 77]. However, both our endothelial aging models show increased transporter (P-gp and Mfsd2a) expression, specifically in the WM of EC-KO mice. P-gp expression can be primarily regulated by inflammation and oxidative stress, as evidenced by increased P-gp levels in stroke and seizure studies [78–80]. Further, other senescent mouse models showed higher P-gp brain vasculature expression, postulating a protective role for senescent cells in toxin efflux from the aging brain [81, 82]. Similarly, the increase in Mfsd2a might also be protective. Mfsd2a limits vesicle-mediated transcytosis, which is crucial to maintain BBB integrity, as shown by barrier leakage in Mfsd2a KO mice [83, 84]. Under homeostatic conditions, increased Mfsd2a expression induces characteristics of cellular aging, while Mfsd2a overexpression alleviates tissue damage after acute brain injury [22, 85, 86]. These evidences highlight the multifaceted role of Mfsd2a in the regulation of EC fitness. It is plausible that the increase of P-gp and Mfsd2a levels in the ERCC1 models is an early protective response to the impaired BBB integrity to aid CNS homeostasis. However, further studies are needed to validate this hypothesis.

The observed changes of the BBB in the EC-KO mice are accompanied by IgG leakage and loss of homeostatic marker expression in microglia in the WM. The leakage of blood-derived components such as fibrinogen has been reported to activate microglia in elderly and AD subjects [87], and to contribute to neuroinflammation and cognitive decline. Of note, while p2ry12 mRNA was increased in the brain of EC-KO mice, its protein expression was decreased in the WM. This discrepancy may be explained by the non-linear relation between mRNA and protein expression, which has been previously reported [88, 89]. We also observed an increase in ICAM1^+^ vessels and leukocyte infiltration, including CD8^+^ T cells, into the WM of EC-KO mice. ICAM1 is a crucial mediator of the immune cell migration cascade and its p53-induced overexpression has been previously found in senescent endothelial cells of atherosclerotic lesions [90]. Moreover, the presence and function of infiltrating peripheral immune cells in the aging brain, AD subjects, and age-related mouse models is increasingly described [91–94]. In the aging brain, CD8^+^ T cells are suspected to interact with CNS cells including microglia and neurons [95], but their role is still heavily debated [96]. Together these results position endothelial cell aging as one potential starting point of BBB inflammation and immune cell infiltration in the aging brain.

Collectively, our data highlight the significance of brain EC aging in BBB dysfunction, a key contributor to the development and progression of several diseases of the CNS. Hence, targeting brain vascular aging may be a promising strategy to alleviate age-related vascular disorders and their neurological implications.

## Supplementary information

Supplementary figure and table legends

Original WB data

Original qPCR data

Supplementary Figure 3. Vascular densities and mural cells in EC-KO and WT brains, related to Figure 4

Supplementary Figure 1.

Supplementary Figure 2.

Supplementary Figure 4.

Supplementary Table 1: Primer details

Supplementary Table 2: FDR-corrected values of multiplex qPCR on shERCC1 and NTC cells

Supplementary Table 3: FDR-corrected values of multiplex qPCR on WBH of WT and EC-KO mice

## Data Availability

Source data are available upon reasonable request.
