# Supplementary material for: Endothelial-Ercc1 DNA repair deficiency provokes blood-brain barrier dysfunction
Source: Cell Death Dis. 2025 Jan 3;16(1):1. doi: 10.1038/s41419-024-07306-0 (PMC11698980; doi:10.1038/s41419-024-07306-0)
Supplement: Supplementary file 1 — Supplementary figure and table legends [file 41419_2024_7306_MOESM1_ESM.docx]

**Endothelial-Ercc1 DNA repair deficiency provokes blood-brain barrier dysfunction**

Cathrin E. Hansen^1,2,3^*^,#^, Davide Vacondio^1,2^*, Lennart van der Molen^1,4^, Annika A. Jüttner^5^, Wing Ka Fung^1^, Manon Karsten^1^, Bert van het Hof^1^, Ruud D. Fontijn^1^, Gijs Kooij^1,2,3,6^, Maarten E. Witte^1,2,3,6^, Anton J.M. Roks^5^, Helga E. de Vries^1,2,3#^, Inge Mulder^2,7,8^^Δ^ , and Nienke de Wit^1,2 Δ,#^

^1^Amsterdam UMC location Vrije Universiteit Amsterdam, Department of Molecular Cell Biology and Immunology, De Boelelaan 1117, Amsterdam, The Netherlands

^2^Amsterdam Neuroscience, Amsterdam UMC, Amsterdam, The Netherlands

^3^MS Center Amsterdam, Amsterdam UMC Location VU Medical Center, Amsterdam, The Netherlands ^4^Radboud university medical center, IQ Health science department, Nijmegen, The Netherlands.

^5^Division of Vascular Medicine and Pharmacology, Department of Internal Medicine, Erasmus Medical Centre, Rotterdam, The Netherlands

^6^Amsterdam institute for Infection and Immunity, Amsterdam UMC, Amsterdam, The Netherlands

^7^Amsterdam UMC location University of Amsterdam, Department of Biomedical Engineering and Physics, Meibergdreef 9, Amsterdam, The Netherlands

^8^Amsterdam Cardiovascular Sciences, Amsterdam UMC, Amsterdam, The Netherlands

*^,Δ^ Both authors contributed equally to this work

^#^ Corresponding authors: CEH (email: c.e.hansen@amsterdamumc.nl, tel: +31 (0) 204448080) and NMdW (email: n.dewit1@amsterdamumc.nl, tel: +31 (0) 204448080)

**Supplementary Figure legends**

**Supplementary Figure 1. ZO-1 expression in shERCC1 and NTC cells and original WB, related to Figure 1**

**a** Representative images of ZO-1 in shERCC1 and NTC cells (scale bar: 25 µm). **b** Original Western blot images of ERCC1 (left) and GAPDH (right) from brain ECs transduced with the ERCC1 knock down construct (shERCC1) or control (NTC).

**Supplementary Figure 2. Mean and maximum length of sprouts, related to Figure 2**

**a** Quantification of mean sprout length, and maximum sprout length in shERCC1 and NTC cells (n=16-20). Each dot represents a biological replicate presented as box plot with median ± quartiles; whiskers extend to minimum and maximum. Statistical comparison of two groups was performed using two-tailed Student’s t-test for normally distributed data, or the Mann-Whitney test for non-normally distributed data.

**Supplementary Figure 3. Vascular densities and mural cells in EC-KO and WT brains, related to Figure 4**

**a** Representative images of LAMININ, PDGFRβ and αSMA immunoreactivity of CRTX and HC in WT and EC-KO mice; white arrowheads indicate αSMA*^+^* vessels (arteriole, upper panel), yellow arrowheads indicate PDGFRβ^+^, αSMA*^-^* vessels (capillary, lower panel) (scale bar: 50 µm). **b** Quantification arterial EC count (percentage of total cells) in WT and EC-KO CRTX, WM and HC brain tissue, (n=6-7). **c** Quantification of vascular densities including overall vessel density and arterial density in WT and EC-KO brain tissue, (n=6-7). **d** Quantification of smooth muscle cell (SMC) count and coverage of arteriole area, (n=6-7). **e** Quantification of MI of PDGFRβ and αSMA in LAMININ^+^ area of WT and EC-KO mice, (n=6-7). **f** Area of IgG reactivity measured in grey matter (GM, average CRTX and HC) tissue of WT and EC-KO mice. Data is presented as box plot with median ± quartiles; whiskers extend to minimum and maximum. All data have been statistically tested by unpaired student-t test with Welch’s correction when the variance of the groups was significantly different.

**Supplementary Figure 4. Astrocyte reactivity in EC-KO and WT brains, related to Figure 5**

**a** Representative images of GFAP in whole brain slices of EC-KO and WT mice (scale bar: 1000µm). **b** Presentation of immune cell subsets (CD45, CD8) in WT mouse brain comparing perivascular and parenchymal location.

**Supplementary Table captions**

**Supplementary Table 1: Primer details**

**Supplementary Table 2: FDR-corrected values of multiplex qPCR on shERCC1 and NTC cells**

**Supplementary Table 3: FDR-corrected values of multiplex qPCR on WBH of WT and EC-KO mice**
