# Supplementary figures and images for: Endothelial-Ercc1 DNA repair deficiency provokes blood-brain barrier dysfunction
Source: Cell Death Dis. 2025 Jan 3;16(1):1. doi: 10.1038/s41419-024-07306-0 (PMC11698980; doi:10.1038/s41419-024-07306-0)

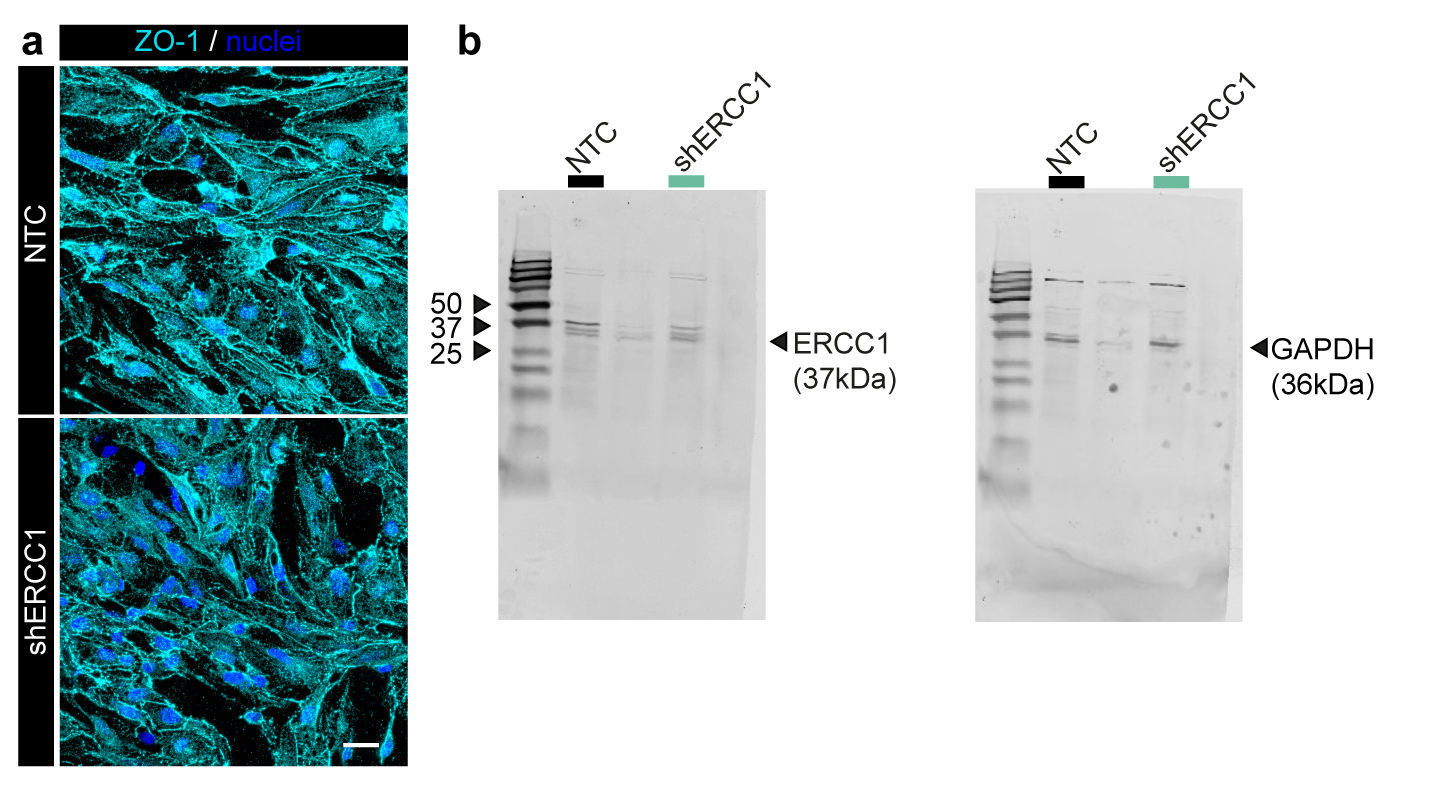

Supplement: Supplementary file 2 — Original WB data [file 41419_2024_7306_MOESM2_ESM.tif]

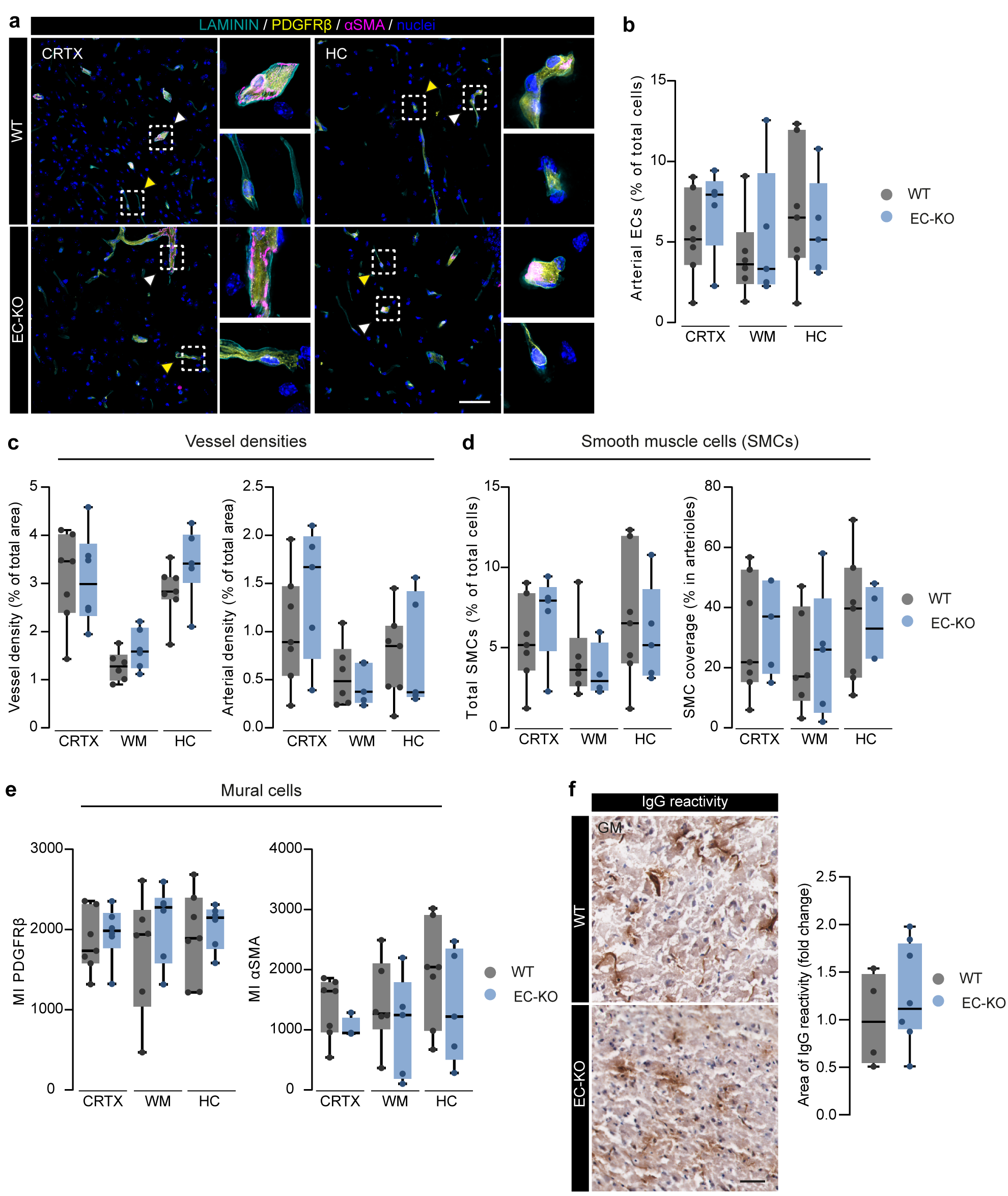

Supplement: Supplementary file 4 — Supplementary Figure 3. Vascular densities and mural cells in EC-KO and WT brains, related to Figure 4 [file 41419_2024_7306_MOESM4_ESM.tif]

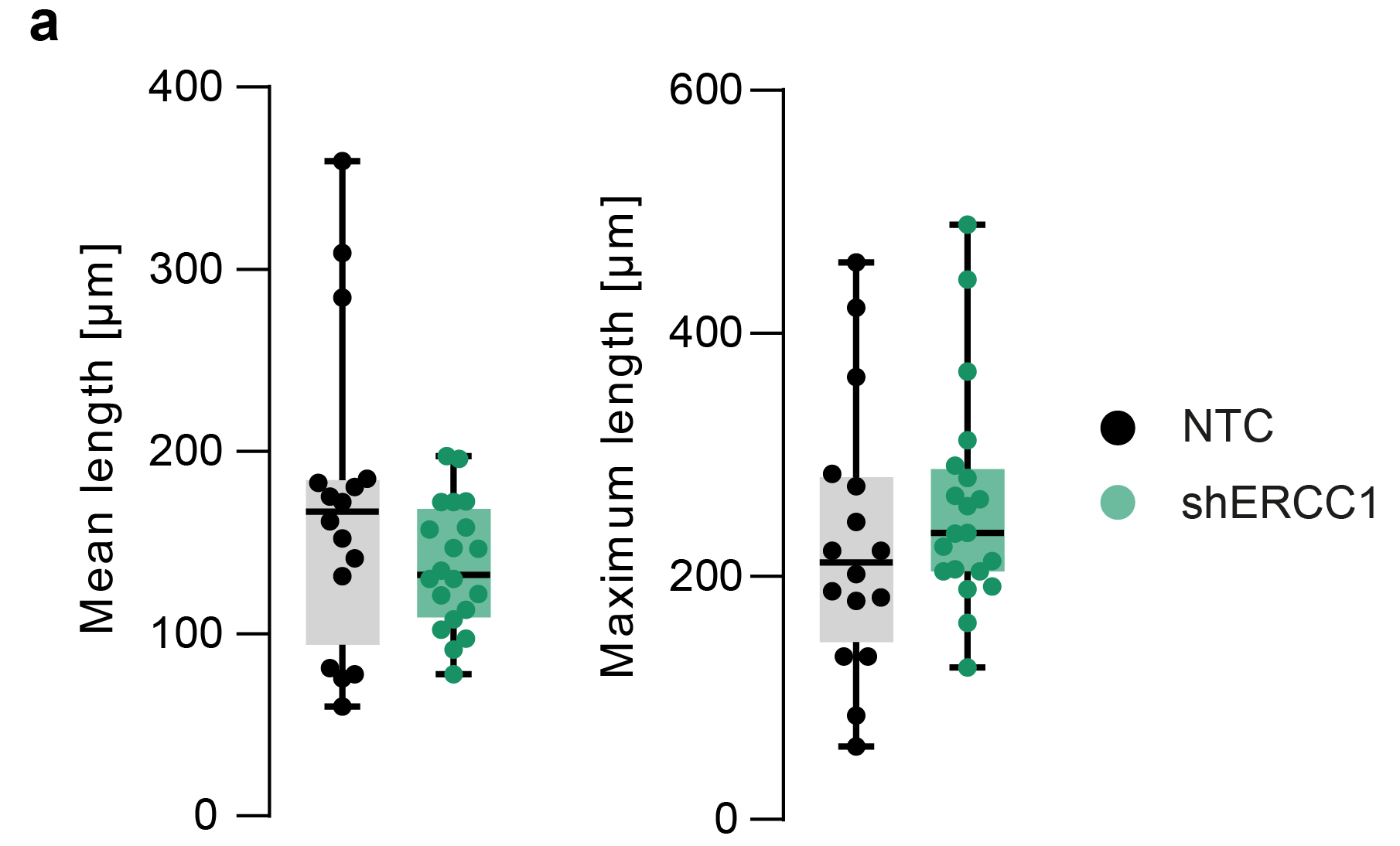

Supplement: Supplementary file 6 — Supplementary Figure 2. [file 41419_2024_7306_MOESM6_ESM.tif]

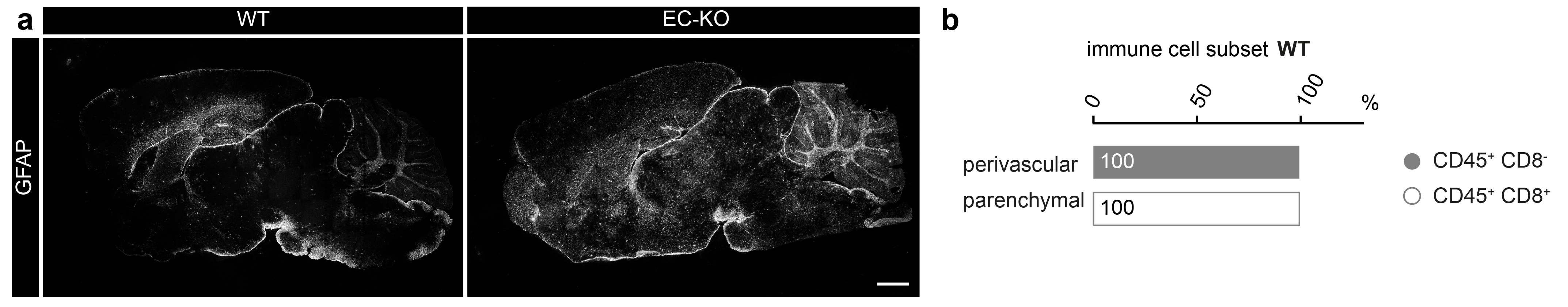

Supplement: Supplementary file 7 — Supplementary Figure 4. [file 41419_2024_7306_MOESM7_ESM.tif]
